# Consumption of Milk Beverages Reduces Iron, Vitamin A, Vitamin C, Calcium, and B Vitamins Inadequacies in Pakistani School-Aged Children from Sindh and Punjab: A Diet Modeling Study

**DOI:** 10.1016/j.cdnut.2024.104435

**Published:** 2024-08-08

**Authors:** Myriam C Afeiche, Diane Zimmermann, Laurence Donato-Capel, Baseer Khan Achakzai, Tsz Ning Mak

**Affiliations:** 1 Nestlé Institute of Health Sciences, Nestlé Research, Société des Produits Nestlé S.A., Vers-Chez-Les-Blanc, Vaud, Switzerland; 2 Nestle Product Technology Center, Societé des Produits Nestlé S.A., Konolfingen, Berne, Switzerland; 3 Health Programs and Regulations, Ministry of National Health Services, Regulation and Coordination, Government of Pakistan; 4 Nestlé Institute of Health Sciences, Nestlé Research, Société des Produits Nestlé S.A., Singapore

**Keywords:** diet modeling, nutrient inadequacy, dairy, milk, school-aged children, Pakistan

## Abstract

**Background:**

Only 47% of households in Pakistan’s Sindh and Punjab provinces are food secure. More than 80% of 5–9 y old children are below recommended intakes of calcium, iron, zinc, and vitamin A and vitamin D. Although 89% consume dairy products daily, only 3% comply with Pakistan’s recommended dairy consumption of 2–3 servings/d.

**Objectives:**

The objectives of this study were to evaluate the theoretical impact of substituting or adding fortified milk and/or buffalo milk in the diets of Pakistani school-aged children to address nutrient inadequacy.

**Methods:**

Dairy consumption and nutrient intakes were calculated using dietary data collected from 5842 children via a 24-h diet recall in the School-age Children Health and Nutrition Survey conducted in Sindh and Punjab provinces of Pakistan between 2019 and 2020. Given daily intakes documented in the School-age Children Health and Nutrition Survey, 2 modeling scenarios were applied to test the impact on nutrient intakes of *1*) substituting current milk (buffalo, cow, and goat) consumption (volume by volume) with a fortified milk beverage and *2*) adding a fortified milk beverage or buffalo milk to current consumption to meet dairy consumption recommendations.

**Results:**

The hypothetical substitution of current milk consumption with fortified milk lowered nutrient inadequacies for vitamin C (by 86%), vitamin A (by 45%), thiamin (by 26%), riboflavin (by 14%), vitamin B6 (by 13%), calcium (by 8%), and iron (by 7%), compared to baseline (relative percent reduction). Among children consuming <2 dairy servings/d, theoretically adding buffalo milk had a positive contribution to calcium, phosphorous, zinc, vitamin A, vitamin C, riboflavin, niacin, and folate; theoretically adding fortified milk additionally reduced inadequacies of iron, thiamin, vitamin B6, and greatly reduced vitamin C inadequacy.

**Conclusions:**

Buffalo milk and fortified milk each have their own value in closing nutrient gaps. Increasing their consumption can be integrated into a multi-pronged public health strategy (including fortified foods, ensuring food security, and diet diversity) to tackle nutrient inadequacies in children.

## Introduction

Nutrition is a key enabler to promote children’s ability to grow, learn, and develop to their full potential. Food security and diet diversity matter to ensure children are provided with adequate nutrient intake. As defined by the WHO, malnutrition, in all its forms, includes undernutrition, inadequate vitamins or minerals, overweight, obesity, and resulting diet-related noncommunicable diseases [1]. Therefore, promoting nutritional solutions to ensure nutrient adequacy for children’s development is key. Among solutions to prevent risk of malnutrition, food fortification is generally recognized as a sustainable long-term approach that is easy to implement [2].

The diet of Pakistani school-age children in Sindh and Punjab provinces is characterized by the consumption of dairy (most commonly milk tea with sugar), bread, sweets, french fries and chips, solid fats, and low consumption of fruits, vegetables, and meats [3,4]. In addition, 68% of households have a high dietary diversity (defined by the consumption of ≥6 food groups in the last 24 h). Only 47% of households in these provinces are food secure. Thus, inadequate micronutrient intakes are prevalent: >75% of children are below the calcium, iron, zinc, vitamin A, and folate recommended intakes. Regarding macronutrients, around 60% of children have inadequate protein intake [lower than the acceptable macronutrient distribution range (AMDR)], around 30% above the AMDR for total fat, whereas most children are within carbohydrate recommendations [3,4].

Milk is a reliable and effective vehicle for fortification purposes due to its widespread consumption and accessibility as a staple in diets, and its consumption is recommended in food-based dietary guidelines [5]. The complexity and nutritional stability of milk make it an ideal vehicle for providing micronutrients that can improve the nutritional quality of diets and thus support normal healthy growth and development in children [6]. Although milk is a good source of calcium and protein, it contains very little iron. Fortified milk, on the contrary, is enriched with additional vitamins and minerals and can thus be a vehicle for missing or inadequate nutrients. Several observational studies and randomized controlled trials found that the consumption of fortified milk-based beverages compared to unfortified milk enhanced nutrient intake (such as iron and vitamin D) in children aged 1 y old and above [7,8]. Diet modeling is a valuable tool to assess the theoretical nutritional impact of how changes to the amounts or types of foods and beverages in a dietary pattern might impact meeting nutrient needs [9]. In line with this, the Irish dietary guidelines, for example, identified that fortified milk contributed to ensuring iron adequacy in toddlers using diet modeling [10].

In this article, we examined how buffalo milk, the most commonly consumed milk in Pakistani school-age children, and fortified milk may contribute to alleviating inadequate nutrient intakes that are key for children’s development. For this, we used diet modeling to first evaluate the theoretical impact of *1*) substituting current milk consumption (volume by volume) with a fortified milk beverage and *2*) adding either buffalo milk or a fortified milk beverage to current intake to meet dairy consumption recommendations.

## Methods

### Study population

The School-age Children Health and Nutrition Survey is a cross-sectional multi-stage household survey conducted over a 6-mo period between 2019 and 2020 in the Sindh and Punjab provinces of Pakistan [3,4]. The survey was commissioned by the Ministries of Health in collaboration with the Trust for Vaccines and Immunizations and Aga Khan University. Demographic, household dietary diversity, child general health, anthropometry, and dietary assessment were collected using structured questionnaires in 5900 school-aged children (5 –<10 y). Further details of the survey and data collection have been previously published [4,5]. Secondary data analysis was conducted using all data from this study. Written consent was obtained from the head of the household prior to data collection, and informed verbal consent was obtained from the children.

### Dietary assessment and data processing

A single multi-pass 24-h diet recall was conducted face-to-face and assessed details of products consumed, their ingredients, and quantity (using standardized utensils such as cups, spoons, bowls, and glasses). Although a second 24 h recall was initially planned, it was not carried out due to the COVID-19 pandemic. Children and their caregivers completed the recall together. Details have been described previously [3,4]. Briefly, foods consumed were converted to nutrient intakes using a food composition database developed for Pakistan in a separate study called “Etiology, Risk Factors and Interactions of Enteric Infections and Malnutrition and the Consequences for Child Health and Development (MAL-ED)” [11]. The MAL-ED database considered the oil fortification with vitamin A but recognized that quality controls suggested variability in content and levels of fortification [12,13]. Thus, oil was considered unfortified with vitamin A. Wheat flour was considered unfortified as the national fortification program was only limited to a few districts in Punjab and was stopped prior to the School-age Children Health and Nutrition Survey. In addition to total energy, analyzed nutrients in the current survey included protein, carbohydrates, total fat, SFA, MUFA, PUFA, calcium, phosphorus, iron, zinc, vitamin A, vitamin D, vitamin C, thiamin, riboflavin, niacin, vitamin B6, and folate. The food composition tables available for local foods did not include vitamin B12 estimates, so vitamin B12 was not part of this study. The nutrient values were summed up at the child level to calculate the intake per child per day. Outliers in energy intakes were identified as above or below 3 standard deviations (SD) of the energy intakes mean in each 1-y age increment (5 y, 6 y, 7 y, 8 y, and 9 y old). From the initial 5900 children in the study [3,4], 58 were excluded due to outliers in energy intake. The final sample size was 5842 children.

Dairy consumption was calculated as the sum of milk (including tea with milk), milkshakes, dairy desserts/custards (such as kheer, sweet vermicelli), yogurt (including lassi), and ice cream as per the FAO Pakistan dietary guidelines [14]. The number of servings of dairy products consumed by each child was calculated to determine adherence to the 2–3 recommended servings of dairy per day, with 1 serving of milk being 250 g [14]. Children who consumed no dairy, or <2 servings/d, were considered not meeting the dairy recommendations. Two types of dairy products were evaluated in the modeling scenarios: buffalo milk (commonly consumed by Pakistani children, either alone or in a sweetened tea beverage) and a reconstituted fortified milk powder (referred to as “fortified milk” from hereon). It is also called filled milk and contains vegetable oils and essential nutrients, including calcium, iron, vitamin A, vitamin C, and vitamin D.

### Modeling scenarios

Two dietary modeling scenarios (substitution and addition) were applied. In the first scenario, we assessed the theoretical impact of substituting current milk consumption with fortified milk among milk consumers (*n* = 4950). In this scenario, we excluded the 89 children (*n* = 1.5%) already consuming fortified milk, resulting in an analytical sample of 4861 children in the substitution scenario. In the second scenario, we evaluated the theoretical impact of adding either buffalo milk or fortified milk to the diets of children consuming less than the dairy recommended consumption per day (2 servings/d) (*n* = 5673). Specifically, current dairy consumption was theoretically topped up with buffalo milk or fortified milk to meet Pakistani recommendations of ≥2 dairy servings per day. For example, in children consuming <0.5 servings/d, we added 2 servings of milk; in children consuming 0.5–0.9 servings/d, we added 1.5 servings of milk; in children consuming 1–1.4 servings/d, we added 1 serving of milk; and in children consuming 1.5–1.9 servings/d, we added 0.5 serving of milk. To address the concern that it may not be feasible/realistic for all families to add ≤2 servings of milk per day, we conducted a third analysis, where we added ≤1 serving/d of milk to children with dairy consumption <2 servings/d. Mean intake and dietary inadequacy were computed before and after modeling scenarios. Results in the text are expressed as relative percent reductions. The composition of buffalo milk and fortified milk used in the modeling scenarios are reported in Supplemental Table 1.

### Data analyses

The estimated energy requirements were calculated using the FAO human energy requirements for children of different ages and sex for moderate physical activity [15]. For all nutrients (except iron and zinc), the 2019 United States dietary reference intakes were used to establish adherence to nutrient recommendations [16] because estimated average requirements (EAR) have not been developed for Pakistan. Previous studies in Pakistan have used similar EARs [3,4]. Children with intake below the EAR for a given nutrient were considered inadequate. For iron and zinc, the WHO EARs were used to account for the low red meat consumption in Pakistani children and, thus, low iron and zinc bioavailability in their diet. EARs for iron and zinc were calculated by dividing the WHO Reference Nutrient Intake (RNI) [17] by conversion factors given in WHO/FAO guidelines on food fortification with micronutrients [18]. For an iron bioavailability of 5%, the WHO RNI is 12.6 mg/d for ages 5–8 y and 17.8 mg/d for age 9 y. Inadequate iron intake was calculated using the full probability method [19]. The EAR for zinc was calculated by dividing the WHO RNI for low zinc bioavailability by the conversion factor. For ages 5–8 y: 9.6/1.2 = 8.0 mg/d; and for age 9 y: 11.2/1.2 = 9.3 mg/d. Given that only 1 d of dietary data was collected, we used the National Cancer Institute SIMPLE macro to adjust for within-person variation to estimate usual intakes and inadequate intakes [20]. Because of the crucial role of selecting an accurate external variance ratio in analyzing single-day data [20], we conducted sensitivity analyses with several variance ratios (ranging from 0.2 to 0.9) on the prevalence of inadequate intakes for 3 selected nutrients (Supplemental Table 2). These external variance ratios were selected according to *1*) a database of publications and reanalyzed datasets (by choosing age groups closest to 5–9 y and South East Asian countries) and *2*) a recommendation to test a range of values from “0.5 to 0.9, or wider, for populations >1 y of age” [21].

## Results

### Sample characteristics

A total of 5842 children were included in this study. Description of the study population has been previously reported elsewhere [3,4]. Although 89.5% of children (*n* = 5231) consumed dairy products, the mean amount consumed per day (150.7 g/d) was <1 serving (250 g/d) (Table 1). The percentage of children classified as dairy consumers did not differ significantly between weekends (89.1%) and weekdays (89.8%), *P* = 0.39. The most consumed dairy product was milk (84.7%, *n* = 4950 children), followed by yogurt (16.9%), ice cream (3.7%), dairy desserts (2.0%), and milkshakes (1.5%). Only 3% of children adhered to Pakistan dairy recommendations of more or equal to 2 servings/d, 17.8% consumed between 1–1.9 servings/d, and 68.7% <1 servings/d (results not shown). The main nutrient inadequacies were vitamin D, iron, zinc, calcium, vitamin A, folate, and vitamin C [3,4].

**TABLE 1:** Dairy consumption in 5–9 y old Pakistani children (*n* = 5842) from the School-age Children Health and Nutrition Survey.

	*n* (% consuming)	Amount consumed, g/d		Mean (SD) amount consumed, servings/day
		Mean (SD)	Max	
Total dairy products	5227 (90%)	145.1 (137)	1500	0.6 (0.5)
All types of milk	4947 (85%)	110.0 (109)	1500	0.4 (0.4)
Buffalo milk	3368 (58%)	135 (120)	1054	0.3 (0.4)
Cow milk	1320 (23%)	140 (108)	947	0.3 (0.4)
Goat milk	171 (3%)	93 (39)	268	0.04 (0.1)
Fortified milk	89 (1.5%)	206 (215)	1500	0.5 (0.6)
Yogurt	986 (17%)	26.5 (75.5)	900	0.1 (0.3)
Ice cream	214 (4%)	2.5 (15.8)	520	0.0 (0.1)
Dairy dessert	119 (2%)	2.8 (23.0)	420	0.0 (0.1)
Milkshake	87 (1.5%)	3.3 (29.4)	600	0.0 (0.1)

Abbreviations: Max, maximum; SD, standard deviation.

### Substitution scenario

**FIGURE 1:**
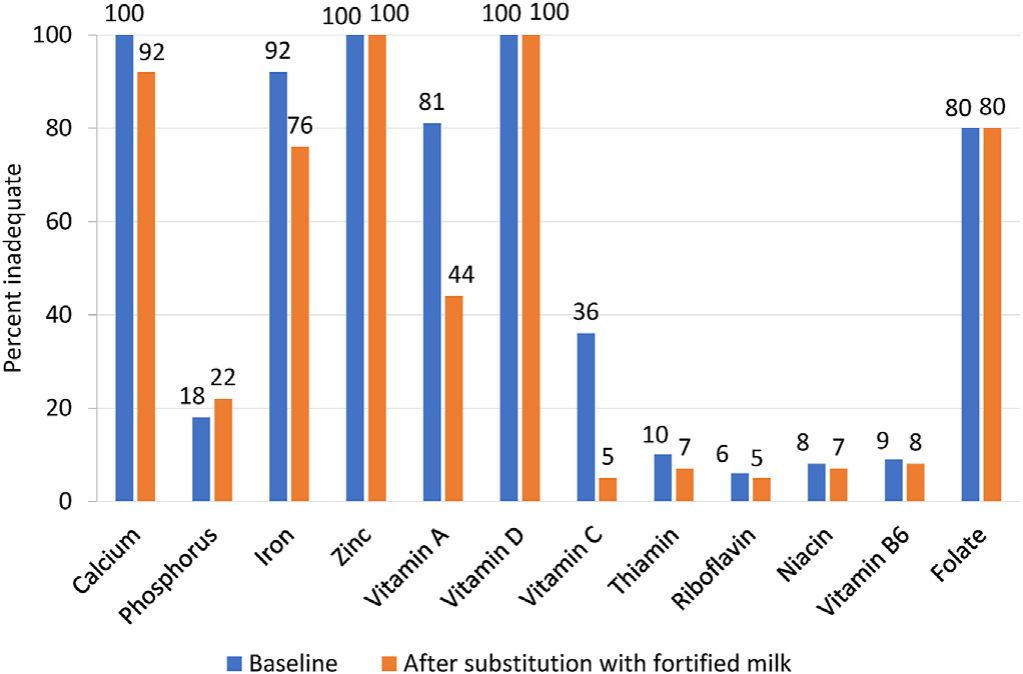
Percent of children 5–9 y old with inadequate nutrient intakes before and after substitution of current milk consumption with fortified milk from the School-age Children Health and Nutrition Survey in Pakistan (*n* = 4861).

Among children consuming milk and excluding children already consuming fortified milk (*n* = 4861 children, 83.1%), substituting current milk consumption with fortified milk did not significantly influence carbohydrate intake (99% compared with 98% of children within the AMDR), but slightly reduced the percent children within the AMDR for protein intake (43% compared with 37% of children within the AMDR) and increased the percent children within the AMDR for fat (62% compared with 74%) (Table 2). The hypothetical substitution of fortified milk instead of milk had the largest impact on lowering nutrient inadequacies for vitamin C (by 86%), vitamin A (by 45%), thiamin (by 26%), riboflavin (by 14%), vitamin B6 (by 13%), calcium (by 8%), and iron (by 7%) (Figure 1). Although mean intakes of vitamin D nearly doubled from 1.0 μg/d to 2.1 μg/d, inadequate intakes remained high at 100%. For phosphorus, however, the percentage of milk consumers with inadequate intakes increased by 24% after the theoretical substitution with fortified milk.

**TABLE 2:** Mean nutrient intakes and inadequacy at baseline and after substitution, in Pakistani children aged 5–9 y (*n* = 4861) from the School-age Children Health and Nutrition Survey.

	Dietary reference intakes^1^		Baseline				After substitution with fortified milk			
	EER or EARor AMDR	UL	Mean	SE	% <AMDRor <EAR	% >AMDRor >UL	Mean	SE	% <AMDRor <EAR	% >AMDRor >UL
Energy, kcal	1399	—	1283	6.8	—	—	1370	8.0	—	—
Protein, g	0.76	—	31.8	0.2	0	—	34	0.3	0	—
Carbohydrates, g	100	—	181	0.7	0	—	195	0.8	0	—
Fat, g	—	—	50	0.4	—	—	51	0.5	—	—
Saturated fat, g	—	—	20	0.2	—	—	21	0.2	—	—
MUFA, g	—	—	13.9	0.16	—	—	13	0.2	—	—
PUFA, g	—	—	7.1	0.1	—	—	7.0	0.1	—	—
Protein, %E	10–30	—	9.9	0.0	57	0	10	0.0	64	0
Carbohydrates, %E	45–65	—	57	0.2	0	1	58	0.2	0	2
Total Fat, %E	25–35	—	34	0.2	0	38	33	0.1	0	25
Saturated fat, %E	—	—	14	0.1	—	—	13	0.1	—	—
MUFA, %E	—	—	9.6	0.1	—	—	8.2	0.1	—	—
PUFA, %E	—	—	4.8	0.1	—	—	4.5	0.1	—	—
Calcium, mg	800; 1100	2500; 3000	371	3.8	100	0	543	7.6	92	0
Phosphorus, mg	405; 1055	3000; 4000	649	3.5	18	0	572	3.4	22	0
Iron, mg	12.6; 17.8	40	6.6	0.1	92	0	10	0.1	76	0
Zinc, mg	8.0; 9.3	12; 23	4.8	0.0	100	0	5.2	0.0	100	0
Vitamin A, μg RAE^2^	275; 420 F 445 M	900; 1700	222	4.7	81	0	341	6.7	44	0
Vitamin D, μg	10	75; 100	1.0	0.04	100	0	2.1	0.0	100	0
Vitamin C, mg	22; 39	650; 1200	43	2.2	36	0	69	2.3	5	0
Thiamin, mg	0.5; 0.7	—	0.8	0.0	10	—	0.8	0.0	7	—
Riboflavin, mg	0.5; 0.8	—	0.98	0.0	6.0	—	1.06	0.0	5.2	—
Niacin, mg	6; 9	15; 20	10.0	0.1	7.6	1.4	10.0	0.1	7.3	1.5
Vitamin B6, mg	0.5; 0.8	40; 60	1.0	0.0	9	0	1.0	0.0	8	0
Folate, μg DFE^3^	160; 250	400; 600	137	1.5	80	0	137	1.6	80	0

Abbreviations: AMDR, acceptable macronutrient distribution range; DFE, dietary folate equivalent; DRI, dietary reference intake; EAR, estimated average requirement; EER, estimated energy requirements; F, female; M, male; MUFA, monounsaturated fatty acid; PUFA, polyunsaturated fatty acid; RAE, retinol activity equivalent; SE, standard error; UL, upper intake level; %E, percent of energy.

1 Numbers separated by a semi-colon refer to DRIs for children ages 5–8 y and 9 y, respectively, except for iron and zinc, where the DRIs refer to children ages 5–6 y and 7–9 y, respectively.

2 Unlike the EAR, the UL is applied to preformed retinol.

3 The EAR for folate is applied to DFEs, whereas the Tolerable UL is applied to folic acid (i.e., from supplements or fortified foods).

### Addition scenario of ≤2 servings/d in children not meeting dairy recommendations

**FIGURE 2:**
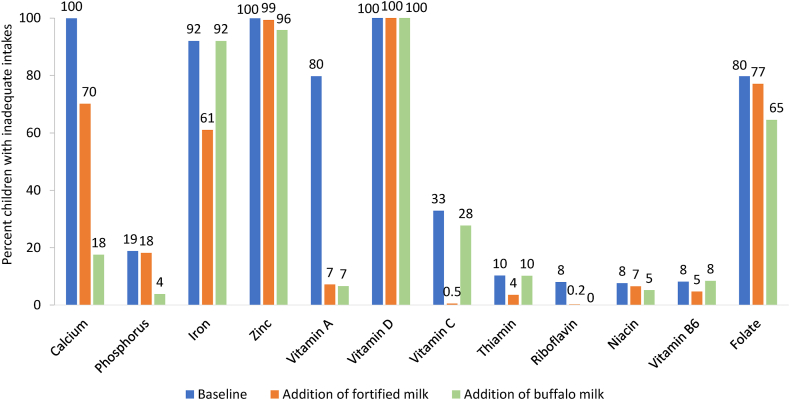
Percent of children 5–9 y old consuming <2 servings/d of dairy with inadequate nutrient intakes before and after the addition of ≤2 portions of fortified milk or 2 portions of buffalo milk from the School-age Children Health and Nutrition Survey in Pakistan (*n* = 5673).

Among children consuming <2 servings of dairy per day (97%, *n* = 5673), with the addition of either fortified milk or buffalo milk, mean energy intakes theoretically increased by 18% and 32%, respectively, and most micronutrient intakes (except vitamin D) were brought closer to the EARs compared to baseline (Table 3). The hypothetical addition of buffalo milk contributed to better macronutrient balance [as a percentage of energy (%E)] for protein. Specifically, adding buffalo milk increased the percentage of children within AMDR for protein from 44% at baseline to 99% compared with 67% when adding fortified milk. However, buffalo milk notably increased the percentage of children above AMDR for fat (from 38% to 100%), whereas fortified milk only slightly increased the percentage of children above the AMDR for fat (from 38% to 49%). Theoretically, adding buffalo milk had a positive contribution to reducing inadequacies for riboflavin (by 100%), vitamin A (by 92%), calcium (by 82%), phosphorous (by 80%), niacin (32%), folate (by 19%), vitamin C (by 16%) and zinc (by 4%) (Figure 2). Similar to buffalo milk, fortified milk theoretically reduced nutrient inadequacies for riboflavin (by 97%), vitamin A (by 91%), calcium (by 30%), phosphorous (by 3%), niacin (14%), folate (by 3%), vitamin C (by 98%), and zinc (by 1%) (relative percent reductions). However, theoretically adding fortified milk furthermore reduced inadequate intakes of iron (by 20%), thiamin (by 65%), and vitamin B6 (by 42%).

**TABLE 3:** Mean nutrient intakes and inadequacy at baseline and after addition of ≤2 portions fortified milk or 2 portions of buffalo milk in Pakistani children aged 5–9 y consuming <2 servings/d of dairy (*n* = 5673) from the School-age Children Health and Nutrition Survey.

	Dietary reference intakes^1^		Baseline				Addition of fortified milk				Addition of buffalo milk			
	EER or EAR or AMDR	UL	Mean	SE	% <AMDRor <EAR	% >AMDRor >UL	Mean	SE	% <AMDRor <EAR	% >AMDRor >UL	Mean	SE	% <AMDRor <EAR	% >AMDRor >UL
Energy, kcal	1404	—	1256	6.8	64	36	1488	6	36	64	1663	6	20	80
Protein, g	0.76	—	31	0.2	0.0	—	38	0	0	—	48	0	0	—
Carbohydrates, g	100	—	176	0.8	0.1	—	205	1	0	—	200	1	0	—
Fat, g	—	—	49	0.4	—	—	58	0	—	—	76	0	—	—
Saturated fat, g	—	—	19	0.2	—	—	25	0	—	—	36	0	—	—
MUFA, g	—	—	14	0.2	—	—	14	0	—	—	22	0.1	—	—
PUFA, g	—	—	7.0	0.1	—	—	7	0	—	—	8	0.1	—	—
Protein, %E	10–30	—	10	0.03	56	0	10	0	33	0	12	0.0	1.4	0
Carbohydrates, %E	45–65	—	57	0.2	0.1	1.4	55	0	0	0	57	0.2	0	0
Total Fat, %E	25–35	—	34	0.1	0.1	38	35	0	0	49	41	0.1	0	100
Saturated fat, %E	—	—	14	0.1	—	—	15	0.06	—	—	20	0.1	—	—
MUFA, %E	—	—	9.5	0.1	—	—	8.0	0.06	—	—	12	0.0	—	—
PUFA, %E	—	—	4.8	0.1	—	—	4.1	0.05	—	—	4	0.0	—	—
Calcium, mg	800; 1100	2500; 3000	345	3.8	100	0	781	2	70	0	958	1.6	18	0
Phosphorus, mg	405; 1055	3000; 4000	629	3.7	19	0	645	3	18	0	1132	2.2	4	0
Iron, mg	12.6; 17.8	40	6.6	0	92	0	13	0	61	0	7	0.0	92	0
Zinc, mg	8.0; 9.3	12; 23	4.7	0	100	0	5.9	0	99	0	7	0.0	96	0
Vitamin A, μg RAE^2^	275; 420 F 445 M	900; 1700	219	5.0	80	0	461	4	7	0	467	4.4	7	0
Vitamin D, μg	10	75; 100	1.0	0	100	0	2.8	0	100	0	1	0.0	100	0
Vitamin C, mg	22; 39	650; 1200	48	3.0	33	0	87	3	1	0	44	2.4	28	0
Thiamin, mg	0.5; 0.7	—	0.8	0	10	—	0.9	0	4	—	1	0.0	10	—
Riboflavin, mg	0.5; 0.8	—	1.0	0	8	—	1.3	0	0	—	2	0.0	0	—
Niacin, mg	6; 9	15; 20	10	0.1	8	1.5	10	0	7	2	11	0.1	5	2.4
Vitamin B6, mg	0.5; 0.8	40; 60	1.1	0	8	0	1.2	0	5	0	1	0.0	8	0
Folate, μg DFE^3^	160; 250	400; 600	137	1.4	80	0	141	1	77	0	157	1.31	65	0

Abbreviations: AMDR, acceptable macronutrient distribution range; DFE, dietary folate equivalent; DRI, dietary reference intake; EAR, estimated average requirement; EER, estimated energy requirements; F, female; M, male; MUFA, monounsaturated fatty acid; PUFA, polyunsaturated fatty acid; RAE, retinol activity equivalent; SE, standard error; UL, upper intake level; %E, percent of energy.

The sample size is 5673 except for EER, where *n* = 5366 due to some children missing weight status.

1 Numbers separated by a semi-colon refer to the DRIs for children ages 5–8 y and 9 y, respectively, except for iron and zinc, where the DRIs refer to children ages 5–6 y and 7–9 y, respectively.

2 Unlike the EAR, the UL is applied to preformed retinol.

3 The EARs for folate are applied to DFEs, whereas the Tolerable UL is applied to folic acid (i.e., from supplements or fortified foods).

### Addition scenario of ≤1 serving/d in children not meeting dairy recommendations

To address the concern that it may not be feasible/realistic for all children to increase milk consumption by 2 servings/d, we restricted the analysis to add ≤1 serving/ d of either fortified milk or buffalo milk in children with dairy consumption <2 servings/d (97%, *n* = 5673) (Supplemental Table 3). A better macronutrient distribution for protein was achieved with the addition of buffalo milk compared to fortified milk and baseline (89%, 56%, and 44% within the AMDR, respectively). Similar to the addition scenario of ≤2 servings/d, the theoretical addition of ≤1 serving/d led to a better fat intake as %E for fortified milk (57% with AMDR) compared with buffalo milk (6% within the AMDR) and comparable to baseline (62% within AMDR). Theoretically, adding buffalo milk had a positive contribution to reducing inadequacies for riboflavin (by 99%), vitamin A (by 65%), niacin (19%), phosphorous (by 16%), folate (by 10%), and calcium (by 8%). Fortified milk reduced nutrient inadequacies for riboflavin (by 79%), vitamin A (by 64%), niacin (8%), phosphorous (by 1%), folate (by 2%), and calcium (by 1%). However, theoretically, adding fortified milk furthermore reduced inadequate intakes of vitamin C (by 90%), thiamin (by 45%), vitamin B6 (by 26%), and iron (by 2%).

### Sensitivity analyses to assess the prevalence of inadequate intakes using different variance ratios

For calcium, percent inadequate intakes ranged from 85% for a variance ratio of 0.2–98% for a variance of 0.9 (Supplemental Table 2). For vitamin C, the percentage of inadequate intakes ranged from 23% for a variance ratio of 0.2 to 1% for a variance ratio of 0.9. Finally, for iron, inadequate intakes were quite stable: 75% for a variance ratio of 0.2 to 73% for a variance ratio of 0.9. The external variance ratio estimate chosen for all nutrients in TABLE 2, TABLE 3 was 0.73. It was selected from a database of publications and reanalyzed datasets by choosing age groups closest to 5–9 y and the geographical proximity of Bangladesh.

## Discussion

The present study explored the theoretical impact of *1*) substituting current milk consumption with fortified milk and *2*) adding fortified milk or buffalo milk to reach the 2 servings/d dairy recommendations on nutrient intakes and adequacy in Pakistani school-aged children. The hypothetical substitution of current milk consumption with fortified milk increased energy and macronutrient intakes and lowered nutrient inadequacies for vitamin C, vitamin A, iron, calcium, and B vitamins, compared to baseline. In children consuming <2 dairy servings per day, the addition of fortified milk contributed to better macronutrient balance (as %E) for fat but not protein as compared to buffalo milk. Theoretically, adding buffalo milk had a larger contribution to phosphorous, calcium, folate, and zinc compared to fortified milk. Whereas the theoretical addition of fortified milk to current dairy consumption had larger reductions in vitamin C, iron, thiamin, and vitamin B6 inadequacies. Thus, buffalo milk and fortified milk each had their own value in closing different nutrient gaps.

Although most children included in this diet modeling study consumed dairy products (89.5%), a large majority (97%) did not meet the dairy recommendations of 2 servings/ d. Milk was commonly consumed (by ∼85% of children), but the mean amount consumed per day was low, less than half a serving per day (110 g/d). Similarly, for yogurt, the mean intake was equivalent to less than one-fifth of a standard yogurt serving size (26.5g/d). Overall, we found that adding milk-based products to the diet of these school-aged children would make a significant contribution to improving their intake of micronutrients crucially meaningful for their growth and development, including the normal function of their immune system (e.g., folate, iron, zinc, vitamin A, vitamin B6, vitamin C, and vitamin D), normal cognitive functions (e.g., iron, zinc), and normal bone growth and development (e.g., calcium, vitamin D, phosphorus, and protein). However, macronutrients were not yet fully balanced, and inadequate nutrient intakes were still high even after substitution or addition scenarios. Thus, increasing milk consumption could be one of the strategies to tackle nutrient inadequacies among Pakistani school-aged children. In Punjab, 44.3% of households are food insecure, and 62.6% in Sindh [3,4]. Thus, a multi-pronged public health plan is needed and could include the consumption of other fortified foods, improving household food security, and ensuring diet diversity, in line with the Pakistan multi-sectoral nutrition strategy 2018–2025 [22]. The development of a school health and nutrition program in the country could be a vehicle for this strategy.

This strategy can be exemplified with vitamin D and iron. Mean intakes of vitamin D more than doubled (from 1 μg/d to 2.1 μg/d in the substitution scenario and from 1.0 μg/d to 2.8 μg/d in the addition scenario with fortified milk); however, virtually all children (100%) remained below the EAR, suggesting that even among children consuming milk, adequate intakes in vitamin D are difficult to reach without further dietary intervention. It should be, however, noted that the contribution to overall vitamin D concentrations, i.e., also including endogenous synthesis through sun exposure, is not taken into account. It is highly acknowledged that the prevalence of vitamin D deficiency is usually high in the pediatric (and adult) population of developing countries [23]. A recent review indicated that 64.6% of healthy individuals in Pakistan had vitamin D concentrations <30 ng/mL [24]. Likewise, the 2018 Pakistan National Nutrition Survey (2018) found a high prevalence (62.7%) of vitamin D deficiency among children below the age of 5, with a significant proportion of them (13.2%) having a severe deficiency [25]. Other studies conducted in Pakistan have reported a high prevalence of vitamin D deficiency in neonates (46–75%), infants (61%), toddlers, and preschool children (17–73%) [23,[26], [27], [28]]. Based on these findings, a “nature and nurture” strategy (i.e., sunlight exposure and supplementation) has been proposed [29].

Iron intake in Pakistan has a low bioavailability, as red meat consumption in children is low [3,4]. Other than chicken and eggs, the main source of iron is from plant-based foods such as lentils, split peas, and chickpeas. Consumption of a fortified milk beverage would typically help to reduce inadequate intakes of nutrients that are not typically found in buffalo, cow, and goat milk regularly consumed by children in Pakistan, such as iron, vitamin C, vitamin D, and some B vitamins which are key nutrients of concern in this population, offering an alternative to those not consuming milk and dairy on a regular basis. In addition, vitamin C consumption would increase the absorption of iron. Although wheat flour has been fortified with iron and folic acid in Pakistan since 2011 [22], consumption of meat and meat products, which have a higher iron and zinc bioavailability, could be encouraged in accordance with Pakistani dietary guidelines [14].

The current study used dietary modeling to test the impact of meeting dairy recommendations to reduce nutrient inadequacy. Like for any dietary modeling study, the data are to be interpreted with caution because they represent a best-case scenario, assuming all children would comply with the recommendations with no compensation or adjustment to the rest of the diet. Another limitation is the use of a single 24 h recall to classify children as dairy consumers compared with nonconsumers. However, most children in Pakistan consume dairy products, and the percentage consuming dairy did not significantly differ by weekend compared with weekday. Thus, we do not expect a within-person variation of dairy by day of the week. Because a single 24 h recall was collected, an external variance ratio had to be selected to calculate the usual intake. Although we conducted sensitivity analyses to determine the robustness of usual nutrient intake distributions and inadequate intake prevalence, it is important to acknowledge that a degree of uncertainty could have been introduced that could have affected the accuracy of results. Finally, given the limitations of back-calculating the EAR from the WHO RNIs [30], the use of different nutrient reference values may lead to differences in the interpretation of nutrient inadequacy. However, the choice of dietary reference intakes for iron and zinc was thoroughly investigated during the data analysis phase according to the differing bioavailability of these nutrients in the diet of Pakistani children. The strengths of this modeling exercise include the availability of a recent and large sample of dietary intake data specific to school-aged children in Pakistan and the calculation of usual intake using one 24 h recall. Lastly, the scenarios are plausible given that the price of fortified milk is comparable to that of buffalo and cow milk.

In conclusion, both buffalo and fortified milk have their own value in closing nutrient gaps. Increasing the consumption of milk among school-aged children should be encouraged to reduce nutrient inadequacy as part of a multi-pronged public health strategy. This strategy could be implemented within a school health and nutrition program in the country and would additionally include the consumption of other fortified foods, improving household food security, and ensuring diet diversity to tackle nutrient inadequacies among Pakistani school-aged children.

## Author contributions

The authors’ responsibilities were as follows – MCA, TNM: designed research; MCA: conducted research and analyzed data; MCA, DZ, LD-C: wrote the article; MCA, DZ, LD-C, TNM, BKA: interpreted results; TNM, BKA: provided critical revisions to the manuscript; MCA: had primary responsibility for final content, and all authors: read and approved the final manuscript.

## Conflict of interest

MCA, DZ, LD-C, and TNM are employees of Nestlé Research, Société des Produits Nestlé S.A. All other authors report no conflicts of interest.

## Funding

This research was funded by Nestlé Research, Société des Produits Nestlé S.A.

## Data availability

Data for specific scientific use can be made available upon request to the Mother & Child Care & Research Inc. (MCCR)
