# Supplementary material for: Consumption of Milk Beverages Reduces Iron, Vitamin A, Vitamin C, Calcium, and B Vitamins Inadequacies in Pakistani School-Aged Children from Sindh and Punjab: A Diet Modeling Study
Source: Curr Dev Nutr. 2024 Aug 8;8(9):104435. doi: 10.1016/j.cdnut.2024.104435 (PMC11421227; doi:10.1016/j.cdnut.2024.104435)
Supplement: multimedia component 1 [file mmc1.docx]

**Supplemental Materials**

**Supplemental Table 1: Composition of one serving (250ml) of commonly consumed milk beverages in Pakistani children participating in the School-age Children Health & Nutrition Survey (SCANS)**

|  | Buffalo milk, 250ml | Fortified milk, 250ml ^1^ | Cow milk, 250ml | Goat milk, 250ml |
| --- | --- | --- | --- | --- |
| Energy, kcal | 247.5 | 144.4 | 165.0 | 172.5 |
| Protein, g | 10.35 | 4.6 | 8.0 | 9.0 |
| Carbohydrate, g | 14.8 | 17.4 | 12.0 | 11.3 |
| Total fat, g | 16.8 | 6.3 | 9.8 | 10.3 |
| Saturated fat, g | 10.3 | 3.5 | 6.0 | 6.8 |
| MUFA, g | 4.8 | 0.2 | 2.8 | 2.8 |
| PUFA, g | 0.5 | 0.1 | 0.3 | 0.3 |
| Calcium, mg | 372.5 | 268.2 | 287.5 | 335.0 |
| Phosphorus, mg | 305 | 17.6 | 230.0 | 277.5 |
| Iron, mg | 0 | 3.6 | 0.3 | 0.3 |
| Zinc, mg | 1.35 | 0.7 | 1.0 | 0.8 |
| Vitamin A, μg RAE | 152.5 | 149.3 | 137.5 | 140.0 |
| Vitamin D, μg | 0 | 1.1 | 0.0 | 0.0 |
| Vitamin C, mg | 2.3 | 23.7 | 2.3 | 2.3 |
| Thiamin, mg | 0 | 0.1 | 0.0 | 0.0 |
| Riboflavin, mg | 0.5 | 0.2 | 0.5 | 0.3 |
| Niacin, mg | 0.3 | 0.1 | 0.3 | 0.8 |
| Vitamin B6, mg | 0 | 0.1 | 0.3 | 0.0 |
| Folate, μg DFE | 12.8 | 3.2 | 12.8 | 2.3 |

^1^ reconstituted milk powder ‘Nestlé Bunyad^TM^’ fortified with essential nutrients including calcium, iron, vitamins A, C and D (so-called ‘fortified milk’).

**Supplemental Table 2: Sensitivity analysis of different variance ratios in calculating the inadequate nutrient intakes for the substitution scenario for 3 selected nutrients**

|  | Proportion of total variance in intake attributable to within-individual variation (WIV:total) | Percent children with inadequate intakes |
| --- | --- | --- |
| Calcium | 0.2 | 85 |
|  | 0.56 | 88 |
|  | 0.73 | 92 |
|  | 0.9 | 98 |
| Vitamin C | 0.2 | 23 |
|  | 0.56 | 11 |
|  | 0.73 | 5 |
|  | 0.9 | 1 |
| Iron | 0.2 | 75 |
|  | 0.56 | 75 |
|  | 0.73 | 76 |
|  | 0.9 | 73 |

**Supplemental Table 3: Mean nutrient intakes and inadequacy at baseline and after addition of up to 1 portion fortified milk or up to 1 portion of buffalo milk in Pakistani children aged 5-9y consuming less than 2 servings/day of dairy from the School-age Children Health & Nutrition Survey (SCANS) (n = 5673).**

|  | **Dietary Reference Intakes ^1,2^** | | **Baseline** | | | | **Addition of fortified milk** | | | | **Addition of buffalo milk** | | | |
| --- | --- | --- | --- | --- | --- | --- | --- | --- | --- | --- | --- | --- | --- | --- |
|  | **EER or EAR or AMDR** | **UL** | **Mean** | **SE** | **% <AMDR or EAR ^1^** | **% >AMDR or >UL ^1^** | **Mean** | **SE** | **% <AMDR or**  **<EAR ^1^** | **% >AMDR or >UL ^1^** | **Mean** | **SE** | **% <AMDR or <EAR ^1^** | **% >AMDR or >UL ^1^** |
| **Energy, kcal** | 1404 |  | 1256 | 6.8 |  |  | 1384 | 6.9 |  |  | 1485 | 6.9 |  |  |
| **Protein, g** | 0.76 g/kg |  | 31 | 0.2 | 0 |  | 35 | 0.20 | 0 |  | 41 | 0.20 | 0 |  |
| **Carbohydrates, g** | 100 |  | 176 | 0.8 | 0.1 |  | 192 | 0.82 | 0 |  | 190 | 0.82 | 0.01 |  |
| **Fat, g** | - |  | 49 | 0.4 |  |  | 54 | 0.42 |  |  | 64 | 0.41 |  |  |
| **Saturated fat, g** | - |  | 19 | 0.2 |  |  | 22 | 0.16 |  |  | 29 | 0.15 |  |  |
| **MUFA, g** | - |  | 14 | 0.2 |  |  | 14 | 0.15 |  |  | 18 | 0.15 |  |  |
| **PUFA, g** | - |  | 7.0 | 0.1 |  |  | 7.0 | 0.11 |  |  | 7.4 | 0.11 |  |  |
| **Protein, %E** | 10-30 %E |  | 10 | 0.03 | 56 | 0 | 10 | 0.02 | 44 | 0 | 11 | 0.02 | 11 | 0 |
| **Carbohydrates, %E** | 45-65 %E |  | 57 | 0.2 | 0.1 | 1.4 | 56 | 0.16 | 0.03 | 0.23 | 51 | 0.14 | 1.81 | 0 |
| **Total Fat, %E** | 25-35 %E |  | 34 | 0.1 | 0.1 | 38 | 35 | 0.13 | 0 | 43 | 39 | 0.11 | 0 | 94 |
| **Saturated fat, %E** | - |  | 14 | 0.1 |  |  | 14 | 0.07 |  |  | 18 | 0.06 |  |  |
| **MUFA, %E** | - |  | 9.5 | 0.1 |  |  | 8.6 | 0.06 |  |  | 11 | 0.05 |  |  |
| **PUFA, %E** | - |  | 4.8 | 0.1 |  |  | 4.4 | 0.05 |  |  | 4.3 | 0.05 |  |  |
| **Calcium, mg** | 800; 1100 | 2500; 3000 | 345 | 3.8 | 100 | 0 | 588 | 3.1 | 99 | 0 | 690 | 3.1 | 92 | 0 |
| **Phosphorus, mg** | 405; 1055 | 3000; 4000 | 629 | 3.7 | 19 | 0 | 632 | 3.3 | 19 | 0 | 913 | 3.3 | 16 | 0 |
| **Iron, mg** | 12.6; 17.8 | 40 | 6.6 | 0.04 | 92 | 0 | 10 | 0.04 | 78 | 0 | 6.6 | 0.04 | 92 | 0 |
| **Zinc, mg** | 8.0; 9.3 | 12; 23 | 4.7 | 0.02 | 100 | 0 | 5.4 | 0.02 | 100 | 0 | 5.9 | 0.02 | 99 | 0 |
| **Vitamin A, μg RAE** ^3^ | 275; 420F 445M | 900; 1700 | 219 | 5.0 | 80 | 0 | 351 | 4.5 | 29 | 0 | 354 | 4.5 | 28 | 0 |
| **Vitamin D, μg** | 10 | 75; 100 | 1.0 | 0.04 | 100 | 0 | 2.0 | 0.03 | 100 | 0 | 1.0 | 0.04 | 100 | 0 |
| **Vitamin C, mg** | 22; 39 | 650; 1200 | 48 | 3.0 | 33 | 0 | 66 | 2.5 | 3.4 | 0 | 43 | 2.5 | 31 | 0 |
| **Thiamin, mg** | 0.5; 0.7 |  | 0.8 | 0.01 | 10 |  | 0.8 | 0.01 | 5.7 |  | 0.8 | 0.01 | 10 |  |
| **Riboflavin, mg** | 0.5; 0.8 |  | 1.0 | 0.01 | 8 |  | 1.1 | 0.01 | 1.7 |  | 1.4 | 0.01 | 0.05 |  |
| **Niacin, mg** | 6; 9 | 15; 20 | 10 | 0.1 | 8 | 1.5 | 10 | 0.06 | 7.0 | 1.68 | 10 | 0.06 | 6.1 | 2.0 |
| **Vitamin B6, mg** | 0.5; 0.8 | 40; 60 | 1.1 | 0.02 | 8 | 0 | 1.1 | 0.03 | 6.0 | 0 | 1.1 | 0.03 | 8.4 | 0 |
| **Folate, μg DFE** ^4^ | 160; 250 | 400; 600 | 137 | 1.4 | 80 | 0 | 139 | 1.4 | 78 | 0 | 148 | 1.3 | 72 | 0 |

^1^ EER: Estimated Energy Requirements. AMDR: Acceptable Macronutrient Distribution Range. EAR: Estimated Average Requirement. UL: Upper Intake Level. %E: percent of energy.

^2^ Numbers separated by a semi-colon refer to DRIs for children ages 5-8y and 9y, respectively, except for iron and zinc where the DRIs refer to children ages 5-6y and 7-9y, respectively.

^3^ RAE: Retinol Activity Equivalents. Unlike the EAR, the UL is applied to preformed retinol.

^4^ DFE: Dietary Folate Equivalents. The EAR for folate is applied to Dietary Folate Equivalents while the Tolerable Upper Intake Level (UL) is applied to folic acid (i.e. from supplements or fortified foods)

Sample size is 5673 except for EER where n= 5366 due to some children missing weight status.
